# Nurse Practitioner: Is It Time to Have a Role in Saudi Arabia?

**DOI:** 10.3390/nursrep10020007

**Published:** 2020-10-09

**Authors:** Hessa A. Almutairi, Kholoud N. Alharbi, Hana K. Alotheimin, Roaa Gassas, Musaad S. Alghamdi, Ayman A. Alamri, Abdulaziz M. Alsufyani, Adel S. Bashatah

**Affiliations:** 1 College of Nursing, King Saud University, Riyadh 84428, Saudi Arabia; 441204104@student.ksu.edu.sa (H.A.A.); 441203335@student.ksu.edu.sa (K.N.A.); 441204176@student.ksu.edu.sa (H.K.A.); 441203341@student.ksu.edu.sa (R.G.); 441106430@student.ksu.edu.sa (M.S.A.); 441106527@student.ksu.edu.sa (A.A.A.); 441105867@student.ksu.edu.sa (A.M.A.); 2 Department of Nursing Administration & Education, College of Nursing, King Saud University, Riyadh 84428, Saudi Arabia

**Keywords:** advanced clinical nurse, advanced practice registered nurse (APRN), cultural barriers, nurse practitioner, nursing shortage, Saudi Arabia

## Abstract

Low recruitment of Saudi nationals into the nursing profession, coupled with a growing population, has led to a severe nursing shortage in Saudi Arabia, particularly of nurses with advanced qualifications in clinical nursing. While the role of nurse practitioner has been successfully integrated into the healthcare systems of the U.S., Canada, the UK and Australia for decades, the advanced practice registered nurse (APRN), which includes nurse practitioners and clinical nursing specialists, is still not being implemented effectively in Saudi Arabia due to a variety of regulatory, institutional and cultural barriers. The author looks at some of those barriers and offers recommendations of how they might be overcome. Given that in many parts of the world, nurse practitioners are considered an essential component to meeting healthcare demands, the author considers the question of whether APRNs can find a role in Saudi Arabia’s healthcare system.

## 1. Introduction

Increasing healthcare costs, growing populations, and a shortage in human resources are creating challenges in meeting the demand for quality patient care worldwide. Saudi Arabia is grappling with a severe shortage of nurses, particularly those with advanced qualifications, due to a host of cultural, educational, and governmental barriers. World Health Organization (WHO) reports also revealed encouragement for advanced nursing practice through its development to cover the global shortage of nurses [1]. Nearly three decades ago, the United Kingdom (UK) reported that senior clinical nurses are equals in providing medical treatment to every individual similar to Physicians. However, in the United States (U.S.), the institute of medicine allowed nurses along with the medical doctors to structure the health care system [2]. Here, we examine the role of nurse practitioners and consider how they might be integrated into Saudi Arabia’s healthcare system in order to increase access to quality patient care.

## 2. Nurse Practitioner Defined

A nurse practitioner (NP) is defined as a nurse with a graduate degree in advanced practice nursing [3]. NPs have at least a master’s degree in nursing, and most have a doctorate degree. NPs can specialize in a specific area, such as pediatrics, oncology, intensive care, or women’s health [4]. NPs have the educational background and experience to provide care and treatment to patients with specific needs [5]. They are essential advocates in the healthcare system and are integrated in many kinds of practice in rural and urban areas, private sectors, hospitals, and nursing home facilities [6]. The NP, the clinical nurse specialist, and other advanced nursing roles fall into the classification of advanced practice registered nurse (APRN). An APRN is defined as a registered nurse who has acquired the expert knowledge base, complex decision-making skills, and clinical competencies for expanded practice [7].

## 3. The Differences between NPs and Registered Nurses (RNs)

Both NPs and registered nurses (RNs) provide care to patients; however, there are differences. NPs are capable of working in similar manner to a physician because they undergo more extensive education and training. RNs can have an associate or bachelor’s degree in nursing, while an NP should have at least a master’s degree in nursing. Having a doctor of nursing practice is suggested for NPs to give them full independence in their practice. Another difference is that over 60% of RNs work in hospitals, while most NPs work in private hospitals or community health clinics. A third difference is the level of autonomy: RNs provide care and administer medication as prescribed by a doctor, while NPs can diagnose and prescribe medicine by themselves [4].

## 4. History of the Nurse Practitioner

NPs first existed in the United States in the mid-1960s at the University of Colorado (UC) to alleviate the shortage of physicians [6]. The NP profession began in response to a dearth of primary care providers, particularly for children, in urban and rural areas in the United States [8]. UC nurse Loretta Ford and physician Henry Silver created the role of NP to improve access to affordable primary care to children and families in underserved communities. They developed a post-baccalaureate pediatric nurse practitioner program designed to increase nurses’ knowledge in the physical and psychosocial development of children, and skills such as performing developmental tests and evaluative procedures, history taking, physical examinations, some laboratory procedures, and referral for medical care that had been the traditional domain of medicine. In 1972, the title of nurse practitioner was adopted by the U.S. Army when it expanded its nurse clinician role [9]. In the mid-1970s, the NP role was resisted by some state medical societies and nursing organizations, and professional relationships between NPs, nurses, doctors, and managers were challenged. Despite this resistance, the NP role continued to grow and evolve [10].

In Canada, the first education program for NPs commenced in 1967 at Dalhousie University in Halifax, Nova Scotia, with the goal of providing care in rural areas. In the 1970s, the Canadian Nurses Association and Canadian Medical Association supported the development of the NP role and university programs [11]. Approximately 250 NPs had graduated and were working as NPs when the movement was suspended in the early 1980s. The NP role reappeared in the 1990s in response to the development of healthcare reform agendas aimed at efficiency and increasing preventative primary care. NPs in Canada have in-depth knowledge and experience in nursing theory and practice, health promotion, disease prevention, and decision-making skills to provide holistic care for individuals [6]. They are legislated, regulated, and permitted to perform comprehensive health assessments, to diagnose and treat health problems, to order and interpret the results of diagnostic and screening tests, and to prescribe drugs and medication. 

In the United Kingdom, NP implementation began in the 1980s, encouraged by cost restraint, a shortage of physicians, and the need to reduce junior doctors’ hours of practice. The University of Birmingham nurse Barbara Stilwell visited the U.S. and Canada to assess NP education and practice and determined that the NP role was appropriate for the UK. In 1990, a formal NP training program was implemented by the Royal College of Nursing (RCN). In 2002, the RCN outlined the domains and core competencies for NPs [10].

In Australia, the growth of the NP role began at a nursing conference in 1990. In 1997, the New South Wales (NSW) Minister for Health worked toward NP authorization, education, and regulation. In 2001, the first NP was appointed to a position in remote NSW, and in 2002, NPs were introduced into metropolitan areas of NSW. NPs must be licensed by a nursing regulatory body to use the title of nurse practitioner. The level of education required for NP authorization varies across Australia, although the trend is toward master’s level [10].

## 5. The Value of the NP Role

Nurse practitioners play an important role in increasing access to healthcare services, especially for people with complex health conditions [6]. They have been nurturing their profession by reflecting on the needs of society and, historically, have been available for those in need of healthcare, offering primary care to the marginalized [6].

According to Chow (2017), nurse practitioners have extensive roles, including assisting patients in decision-making, promoting patient and family engagement, providing continuity of care, improving patient satisfaction, improving interpersonal communication and professional relationships, decreasing morbidity and mortality rates, increasing treatment compliance, improving productivity, and reducing healthcare costs [12].

The role of nurse practitioner is essential to meeting the current healthcare demand. A study by Buerhaus et al. [13] found that physicians and nurse practitioners provide similar types of services. Yet, although their services are similar, nurse practitioners convey more information to patients regarding health monitoring [14].

## 6. Saudi Arabia’s Nursing Shortage

Achieving and maintaining a stable nursing workforce is an important goal in ensuring the well-being of Saudi Arabia’s rapidly growing population. However, a high turnover of expatriate staff and low recruitment of Saudi nationals have led to a serious shortage in the profession, particularly of well-qualified and experienced nurses [15]. According to 2018 data from the Saudi Ministry of Health (MOH), Saudi nationals represent just 38% of nursing staff. The demand for nurses is high, and the number of nursing students and graduates is too low to meet national needs. A study conducted in 1982 by two faculty members at King Saud University concluded that the number of Saudi men and women entering the nursing and medical professions was far below the national demands [16]. Those findings have borne out as in the past decade, the percentage of Saudi nurses increased by just 9%—from 28% in 2008 to 38% in 2018 [17,18]. As a result, Saudi Arabia continues to rely on foreign nurses to close the gap in the nursing workforce.

In addition to an overall nursing shortage, there is a severe shortage of nurses with advanced qualifications in clinical nursing. Although several studies have shown the importance of APRNs in the healthcare system, advanced nursing practice is still not implemented effectively in Saudi Arabia, except in one tertiary hospital (King Faisal Specialist Hospital and Research Centre) which hires experienced expatriates for this role [19].

## 7. Barriers to Incorporating APRNs into the Saudi Healthcare System

The World Health Organization (WHO) has acknowledged the need for APRNs in the Middle East, including Saudi Arabia [20]. However, various regulatory, institutional, and cultural barriers restrict the practice of APRNs. According to Hibbert et al. [21], reasons for the APRN shortage include a lack of higher education programs, low pay, and a negative image of clinical nursing in Saudi Arabia. 

## 8. Nursing Education Programs

Nursing education in Saudi Arabia started in 1954 with a one-year program. The first bachelor’s degree program in nursing was established in 1976 for women. For men, the bachelor’s degree in nursing began in 2004 [15]. In 1994, the Saudi Commission for Health Specialties (SCFHS) was established to accredit health education programs and to license healthcare professionals.

Although nurse training programs have been offered for 40 years, having transitioned from hospital-based education to college baccalaureate and master’s programs, the postgraduate university education aimed at the advancement to APRN is limited [22]. Master’s programs in nursing are few, and most are Master of Science, with a curriculum based in research and a focus on nursing administration and education. 

In line with WHO recommendations, a higher degree for nursing was recently established in a small number of universities in Saudi Arabia. The SCHS established a postgraduate two-year diploma aimed at developing specialized nursing skills in the areas of emergency nursing, cardiology, critical care, oncology, neonatology, intensive care, and home healthcare [23]. However, graduates from these programs do not earn a master’s degree that would qualify them to be a candidate for NP.

Two new programs are now being offered in clinical practice: one in critical care nursing and the other in advanced nursing practice (ANP). However, although both programs focus on clinical practice, the degree is still called Master of Science in Nursing, even though the literature suggested that there is a chronic shortage of nurses and nurse practitioners in Saudi Arabia, similar to other countries [24,25]. The shortage of this profession is mainly due to the majority of Saudi hospitals being occupied by foreign health care professionals; once these nurses are experienced in their current setting, they start looking for high paid jobs and move to other European and American countries. In reality, Saudi hospitals depend on an expatriate workforce of nurses (>60%) who come from more than 40 diverse countries; among those, the top listed were the United States, Australia, the Philippines, and India [25].

## 9. Incentive, Recognition, and Licensure

Saudi nurses who obtain advanced clinical nursing qualifications have the same job description as a registered nurse in the clinical setting. There is no differentiation in either job description or incentives between holders of a Master of Nursing degree and those with an ANP degree. 

The MOH does not recognize the advanced nursing practice within the formal healthcare structure [19]. This also applies to nurses who obtained an NP degree from an international school on a government scholarship.

## 10. Legislation and Regulation

The slow progress in recognizing the APRN profession and accrediting an APRN program is due. in part, to a lack of legislation from the Ministries of Education, Health, and Human Resources. Hibbert et al. [19] found that the lack of nursing legislation and regulation in the Saudi Arabian healthcare system limits advanced nursing practice. A 2010 Institute of Medicine report, which identified country regulations that impose restrictions on APRN practice, cited, as examples, the limits of an NP’s ability to admit clients, act as a primary care provider, sign a long-term care order, pay for services, and be accepted by health insurers [26]. Currently, in Saudi Arabia, the legal and regulatory foundations are not in place to permit these activities.

## 11. Health Administration Policy

The lack of advanced nursing practice in Saudi Arabia may also be due to a gap between nursing scientists and the policymakers in the upper level of health administration at healthcare facilities. Examples of deterrents that could be addressed by closing the gap include poor role clarification, proliferation of APRN titles, varying educational requirements and degrees, range of practical conflicts, variability in standards, and quality of education programs [27,28].

## 12. Recommendations

The Saudi Commission for Health Specialties (SCFHS) oversees executive nursing councils, including the Professional Practice Council, the Education and Training Council, and the Nursing Scientific Board (NSB) The NSB formulates regulations and criteria for professional practice and advises the SCFHS on licensing [29]. Policies should be established that would encourage nurses to pursue an NP degree by minimizing negative attitudes, increasing incentives, and ensuring they will be able to practice to the full extent of their capabilities. It is recommended that these nursing bodies collaborate and take the lead in formulating these policies. 

The formulation of policies encouraging advanced nursing practice must happen in partnership with nursing education standardization and nursing profession regulations [19]. Nursing scientists should participate in nursing decisions and policy formulation to achieve desired outcomes. Nursing educators should work within their institutions to address barriers in order to improve the nursing discipline in Saudi Arabia. Nurse managers also have a role by sharing the importance of advanced nursing practice on patient care in healthcare facilities.

## 13. Looking to the Future

Considering the urgent need for nurses in Saudi Arabia, and that an NP must first become an RN, the question could be asked: should the priority be to incorporate NPs into the healthcare system, or to instead focus on filling the RN shortage? To answer this question, various factors, such as cost, length of education, and scope of services of both RNs and NPs, should be considered to determine which would be the most cost-effective and beneficial for patients.

According to the previous report, the employment of RNs is projected to grow 12% between 2018 and 2028, while the demand for NPs will grow by 28% during that same time [30]. In spite of the current barriers, Saudi nurses are optimistic about their future, and it is expected that an increasing number of them will pursue APRN qualifications in the future.

However, the nursing profession in Saudi Arabia is still at an early stage, and the APRN role cannot be established successfully without clear basic rules, regulations, scopes, and definitions. A further requirement will be confidence in the value of and need for such a role in the nation’s healthcare system and a commitment to building the foundation required to take root and thrive.
